# Understanding the facilitators and barriers of antiretroviral adherence in Peru: A qualitative study

**DOI:** 10.1186/1471-2458-10-13

**Published:** 2010-01-13

**Authors:** Walter H Curioso, Deanna Kepka, Robinson Cabello, Patricia Segura, Ann E Kurth

**Affiliations:** 1 Epidemiology, STD/AIDS Unit, School of Public Health and Administration, Universidad Peruana Cayetano Heredia, Honorio Delgado 430, Lima 31, Peru; 2 Division of Biomedical and Health Informatics, Department of Medical Education and Biomedical Informatics, School of Medicine, University of Washington, Seattle, WA 98195, USA; 3 Department of Health Services, School of Public Health, University of Washington, Seattle, WA 98195, USA; 4 Asociación Vía Libre, Paraguay 478, Lima 1, Peru; 5 Asociación Civil Impacta Salud y Educación, Grimaldo del Solar 805, Lima 18, Peru; 6 Biobehavioral Nursing and Health Systems, School of Nursing, University of Washington, Seattle, WA 98195, USA; 7 Current address: New York University College of Nursing, 726 Broadway, 10th Floor, New York, NY 10003, USA

## Abstract

**Background:**

Antiretroviral scale-up is increasing in resource-constrained settings. To date, few studies have explored the barriers and facilitators of adherence to ART in these settings. Facilitators and barriers of antiretroviral adherence in Peru are not completely understood.

**Methods:**

At two clinics that serve a large number of HIV-positive individuals in Lima, Peru, 31 in-depth interviews were carried out in 2006 with adult HIV-positive individuals receiving ART. Purposive sampling was used to recruit the participants. Interviews were transcribed and coded using two Spanish-speaking researchers and a content analysis approach to identify themes in the data.

**Results:**

Among the participants, 28/31 (90%) were male, 25/31 (81%) were self-identified as mestizo, and 19/31 (61%) had an education above high school. The most frequently discussed barriers to adherence included side effects, simply forgetting, inconvenience, dietary requirements, being away from home, and fear of disclosure/stigma. The most frequently discussed facilitators to adherence included having a fixed routine, understanding the need for compliance, seeing positive results, treatment knowledge, and faith in treatment.

**Conclusions:**

Overall, these findings were similar to the facilitators and challenges experienced by individuals on ART in other resource constrained settings. Further treatment support tools and networks should be developed to decrease the challenges of ART adherence for HIV-positive individuals in Lima, Peru.

## Background

Individuals living with HIV/AIDS in resource-constrained settings now have increased access to antiretroviral therapy (ART) medications. In Peru, the HIV/AIDS epidemic has been largely concentrated among men who have sex with men (MSM) [1,2]. The seroprevalence for MSM is 10-22% [2-4], compared to 0.1-0.4% for the general population [5], and 1% for female sex workers [6]. Antiretroviral therapy, available through the national ART program in Peru since 2004 [1,7], is a key component of improving health outcomes for HIV-positive individuals. To achieve optimal results from ART, high levels of patient adherence to ART are critical [8-10]. The availability of ART allows HIV patients to be managed effectively as a serious chronic rather than terminal illness [8,11].

Adherence plays an important role in the effectiveness of ART because individuals who demonstrate lower levels of adherence are at greater risk for increased levels of morbidity, treatment failure, and the development of drug resistant forms of HIV [12-16]. To achieve optimal effectiveness for reducing viral load, patients take 90-95% of their prescribed antiretroviral medications [14]. However, viral suppression may still occur with lower levels of mean adherence, from 54% - 100%, to potent regimens [17]. When viral load rises, risk of transmission increases [18] and the quality and longevity of life decreases [19,20].

Studies have shown that a large percentage of patients, in general, find it difficult to achieve high levels of adherence to a treatment. In a meta-analysis of 569 studies, patients display a non-adherence rate of approximately 25% on average [21]. Non-adherence is particularly difficult for HIV-positive individuals because ART regimens are complicated, have complex side effects, and associated stigma. Past studies have shown that approximately 12-50% of HIV-positive patients fail to achieve optimal adherence [22-27]. In a meta-analysis of adherence to ART that included 37 qualitative studies and 47 quantitative studies in developed and developing nations, Mills et al. (2006) found that many barriers to adherence were reported similarly in a number of settings and countries. Of the 37 qualitative studies that were conducted, only two qualitative studies were carried out in developing countries [28]. In Peru, adherence to HIV risk reduction and treatment is becoming increasingly problematic according to anecdotal reports from local AIDS clinicians.

To provide optimal HIV care, strategically developed interventions that are tailored and multi-focused are needed to improve ART adherence. However, the development of effective and tailored interventions to improve ART adherence requires an in-depth understanding of the mutable factors that influence it. Few qualitative studies have been carried out in resource constrained international settings [28] and there have not been any in-depth qualitative studies conducted in Lima, Peru related to the barriers and facilitators for ART adherence.

The present study describes the social, cultural, knowledge-based, and logistical factors that influence ART adherence in Peru. Heightened knowledge of these specific barriers and facilitators for ART adherence in Peru will aid in the development of useful and needed interventions to promote improved ART adherence in resource constrained settings.

## Methods

Formative research techniques were carried out to assess adherence barriers and facilitators towards ART medication in Lima, Peru. This was done using in-depth interviews with adult HIV-positive people receiving ART and clinical services at two community-based clinics (Impacta and Via Libre). We also addressed access, use, and perceptions of the Internet, cell phones, and PDAs as a means for health promotion that were reported in a separate study [29].

Both of these clinics primarily serve male clients (100% at Impacta, approximately 80% at Via Libre) and demonstrate similar demographic profiles for their patient populations. These two clinics were selected because they are two of the most important clinics for HIV positive individuals in Lima and they have participated in other research studies. Purposive sampling was employed at the two clinics to recruit individuals for study participation [30]. In addition, flyers were posted at the clinics to recruit participants into the study over the course of six months. To qualify for the study, participants had to be at least 18 years old, HIV positive, and receiving ART.

Past studies informed the development of the topic guide for the semi-structured in-depth interviews [31,32]. The topic guide was then reviewed for content validity by HIV consultants in Lima. An outline of questions related to lifestyle, living with HIV, ART medication are listed below:

### Lifestyle

Describe a normal day in your life.

• With whom are you living right now?

• Who are the important people in your life?

• What are your major concerns right now?

### Living with HIV

• When did you first find out you were HIV positive? What was that like for you? Has HIV changed your life?

• Have you told anyone in your life about your HIV status? Why or why not? If yes, what was their reaction?

• What sources of support for living with HIV have you been able to find?

### ART Medication

• What have you heard about medicines that are used to treat HIV/AIDS (antiretrovirals)? How is it that they work in the body?

• What have you heard about 'HIV viral load'? What have you heard about 'CD4' (good immune cells)?

• How well do you think these medicines help people with HIV/AIDS?

• Do you believe these medicines harm people with HIV/AIDS? Why?

• How long have you been on these medicines? How has that been for you? Have you had any problems on these medicines?

• How important do you think it is to take all of your doses of this medication? What happens if people miss doses?

• Has your day-to-day life had to change to take these medicines? How?

There are certain ways people are told to take these medicines. For example, take a certain number of pills at a certain time of day.

• How has it been for you to take these medicines this way?

• What makes it hard to take these medicines? (*cost, transportation, childcare, stigma?)*

• What helps you take these medicines? (*pill boxes, daily routine, reminders, regular meal times, privacy, support of friends or family, not drinking as much alcohol?)*

• How serious is it to have HIV now that medicines to treat people with HIV/AIDS are here?

The in-depth interviews were carried out by an experienced and trained psychologist in each of the clinics (Impacta and Via Libre). After giving consent, all interviews were conducted in Spanish with each respondent in a private room for approximately one hour. Each interview was tape-recorded. All participants signed an informed consent form prior to entry into the study. Participants were compensated with 30 soles (about 10 dollars) for travel expenses.

Audio files were transcribed and transcripts were reviewed by a Spanish-speaking investigator (WC) for initial text element and key word coding. Transcripts were then reviewed separately by two Spanish-speaking authors (WC and DK) for text element and key word coding, and initial generation of themes. The coding of the text into categories of experiences and beliefs resulted in the emergence of thematic concepts. A theme was then defined as a "common thread that runs through the data" [33]. Summaries of coding practices and thematic concepts were compared and discussed over 10 meetings until a consensus was reached on which themes were most salient to participants' responses. We then reviewed the transcripts to confirm our findings until we reached saturation and identified quotes that best illustrate common theme [34]. Quotes were edited for ease of reading but were not substantially altered. Data were entered into Atlas.ti version 5.5 (2008) qualitative data management software (Scientific Software Development, Berlin, Germany) for theme identification using a content analysis approach. The study had ethical approval from the University of Washington Human Subjects Division (HSD# 06-1430-G-01) and the institutional review boards of Via Libre and Impacta.

## Results

### Description of Study Participants

During March-August 2006, 31 people living with HIV were interviewed, 16 at Via Libre Clinic and 15 at Impacta Clinic. Of these participants, 28/31 (90%) were male, 25/31 (81%) were self-identified as mestizo (multi-ethnic and/or multi-racial), and 19/31 (61%) had an education level above high school. All participants were currently on ART.

### ART Adherence Facilitators

In general, participants who were able to describe the effects of the medication appeared to be more conformable with adhering to the regimen. This "information" element has been theorized to be an important component in ART adherence [35] and is an area that health professionals are well qualified to address. For many people, overcoming the side effects of ART was the first stage that must be addressed to achieve high levels of adherence. The second stage for many individuals was successfully incorporating ART into one's every day routine. Participants reported a number of specific factors that influenced improved levels of adherence to ART.

#### Patient Characteristics

First, many factors were identified that relate to individual level patient characteristics that represented facilitators to improved ART adherence. These include seeing positive results (reported by 65% of participants, 20/31), learning to manage (reported by 55% of participants, 17/31), and self-efficacy for high levels of adherence to the ART regimen (reported by 32% of participants, 10/31). Seeing positive results and learning to manage were two of the most frequently discussed adherence facilitators by the participants in this study at the patient characteristics level. Participants who knew that ART was positively impacting their health and those who learned to manage the impact of ART regimens on their everyday lives reported these characteristics as important ART adherence facilitators.

An example of learning to manage is expressed by this participant:

"I made antiretrovirals part of my life. It's true that the pills make me sick, but after some time the [bad sensation/pain] that I felt is something that is not impossible to manage." Male, white, 43 years

Examples of seeing positive results were expressed by these participants:

"The antiretrovirals have helped me to increase my CD4 from 170 to 230 and not only that but also improve my spirit." Male, mestizo, 28 years

"Taking the antiretrovirals helps a lot. I can see for myself: Before I started the treatment, I weighed 52 kilos and now I am at 74 kilos. I believe that the treatment helps because in some way I am seeing the results." Male, white, 48 years

An example of self-efficacy to adhere to ART is expressed by this participant:

"What makes me take my antiretroviral medicines is my spirit. My spirit tells me always that I have to take my pills to feel good with myself." Male, mestizo, 25 years

#### Beliefs about the Medication

Second, many factors were identified that relate to beliefs about the medication that related to improved levels of adherence. These include beliefs in efficacy of drugs (reported by 42% participants, 13/31), faith in treatment (reported by 42% participants, 13/31), and understanding the need for compliance (reported by 97% participants, 30/31). Specifically, understanding the need for compliance was one of the most frequently discussed facilitators to adherence in this study as expressed on one participant:

"If I want to continue living, I have to adhere to the treatment because it is the only way to be healthy. This is like a diabetic patient. If he/she does not take their medicine, he/she will die. It's not because of the sugar but because all that occurs when the sugar increases." Male, white, 48 years

Participants who were aware of the need for compliance to their medication regimens were more likely to cite this factor as key to increasing self-motivation for adherence.

Other participants expressed belief in the efficacy of the drugs and a strong faith in treatment that facilitated motivation for adherence. One participant states:

"Taking my antiretrovirals will make me feel good, I know that I will not be cured, but will help me to live longer." Female, mestizo, 38 years

Another participant expresses:

"Antiretroviral medicines give me the opportunity to continue living, to continue being with my friends and family." Male, white, 43 years

#### Daily Schedules

Third, many factors were identified that relate to daily schedules were associated with strategies for improved levels of adherence. These include having a fixed routine (reported by 71% participants, 22/31), and the use of reminder tools (reported by 58% participants, 18/31). Having a fixed routine was the top adherence facilitator that was discussed by participants as related to daily schedules. As expressed by one participant:

"I have my brain alarm, when it's time to take my antiretroviral pills it starts to buzz... it's eight...time to take your pill." Male, mestizo, 35 years

Many participants reported concrete strategies to successfully integrate their pill taking regimens into their lives as key facilitators to adherence such as the use of reminder tools:

"I have programmed the alarm of my cell phone, with a very nice ring tone. When it rings I know automatically that I have to go where my pills are and take them." Male, mestizo, 36 years

#### Interpersonal Relationships

Lastly, a number of factors were identified that relate to interpersonal relationships that helped improve levels of adherence. These include family and friends reminding the participants to adhere to their ART regimen (reported by 29% participants, 9/31), and living for someone (reported by 16% participants, 5/31). As expressed by two participants:

"My mom or my sister remind me to take my medicines. For example, my sister tells me "have you taken your "contrita" (a popular name for the medicine)? So I respond 'yes, I took my 'contrita'." Male, mestizo, 34 years

"I live for my daughter, for her, I try to get through." Female, mestizo, 38 years

Many participants reported that the support of others, including their health care provider (e.g. doctor, nurse, counsellor), in their lives was an important and effective adherence facilitator. As represented by the following experience noted by a participant:

"My new doctor is more worried about me...we are working together. We have changed my medication and I feel completely relieved." Male, mestizo, 36 years

#### Other Facilitators

Many other ART adherence facilitators that were briefly discussed by participants include the following. Many participants reported positive and open relationships with their medical providers as an important adherence facilitator (reported by 39% participants, 12/31). Feeling comfortable asking questions, talking about challenges, and feeling a part of decision making with one's medical provider played a key role in facilitating adherence. Next, having a simple regimen was reported by a few participants as assisting in ART adherence. Lastly, medication taking priority over substance use, accepting HIV status, and being open with disclosure of HIV status were noted by a few participants as important adherence facilitators.

### ART Adherence Barriers

A wide variety of barriers to ART adherence were discussed by the participants. These were classified into three broad categories: patient characteristics, beliefs about medications, and daily schedules.

#### Patient Characteristics

First, patient characteristics include the following factors: simply forgot (reported by 36% participants, 11/31); fear of disclosure/stigma (reported by 23% participants, 7/31); and financial constraints (reported by 13% participants, 4/31). Simply forgetting one's medication was one of the most frequently cited adherence barriers by the participants within the category of patient characteristics. As represented by this participant's experience:

"I think that forgetting to take your medicines is that you simply miss the time, because you know that you have to follow a scheme, to follow a treatment, but there were moments that I had to take the pill and I missed it." Female, white, 32 years

The participants also discussed difficulties with adhering to medication regimens without disclosing their HIV status to others at work and/or at home. A number of participants touched on this barrier:

"Initially, I took my treatment in a particular way. It was my cross, nobody knew about it, nobody, during five years, nobody, nobody knew about it, only my physician and me." Male, white, 48 years

"I have to hide from my family so they do not know that I'm taking my ART medication. They do not know anything about my disease." Male, mestizo, 36 years

"One time I had a pill box, a hexagon, but it was...very big, and they asked me: hey, 'what are those pills?' 'ah, they are vitamins'." Male, mestizo, 27 years

In addition, financial constraints and fear of disclosure/stigma were also frequently discussed. Specifically, participants noted financial challenges related to acquiring their medications on time. One participant remarks:

"The problem with taking my pills was always economic. At the beginning it was very expensive, then prices went down. Because of this, it is very difficult to maintain an exact frequency for taking my pills and I had holes in the treatment." Male, mestizo, 39 years

#### Beliefs about the Medication

Second, beliefs about the medication were discussed as barriers to ART adherence by the participants. This category included the following factors: side effects (real or anticipated) reported by 74% participants, 23/31; harmful (reported by 19% participants, 6/31); and not convinced of efficacy (reported by 16% participants, 5/31). Overall, side effects were the most frequently discussed barrier to adherence by participants. Side effects included both negative symptoms that the participant attributed to the ART regimen and side effects that were anticipated to occur due to the ART regimen. As highlighted by one participant:

"When I started with the treatment I was nauseous all the time. I had headaches. I had a fever. I had cellulitis again. I experienced a lot of illnesses that I had never had before....I was very very bad off." Male, white, 43 years

#### Daily Schedules

Lastly, daily schedules were reported as additional challenges to ART adherence. This category includes the following factors: dietary requirements difficult to balance (reported by 26% participants, 8/31); being away from home (reported by 23% participants, 7/31); and too busy (reported by 16% participants, 5/31). Specifically, being away from one's household during the course of one's day for work or social engagements was reported as greatly interfering with adherence to the ART regimen. As discussed by three participants:

"I have forgotten to take my pills when I go out in a hurry out of my house or when I had to stay at work. When the time to take my pill passed (8 pm), and I thought: 'oops...my pill...I didn't take it'." Male, mestizo, 35 years

"For me, weekends are terrible because I have to go out to a party or a meeting and I do not take the pill at night. It is a problem for me." Male, mestizo, 28 years

"I have to take my pills with food. This is the problem and what makes me upset is that there are some days that I cannot eat and I cannot take my pills." Male, mestizo, 35 years

A few other participants found the dietary requirements difficult to balance:

"I have to pay attention to my diet and there are some times that I cannot control it. This makes it a little bit difficult to take my pill." Male, mestizo, 36 years

In addition, a number of participants felt that they were too busy to remember their pills. As expressed by one individual: "There are some times that I have so many things in my head that I forget to take my pills." Male, white, 35 years

#### Other Barriers

Other barriers to ART adherence that were briefly discussed by the participants included the following. Some participants lacked trust in their medical provider and felt that this was a barrier to adherence. Some were feeling healthy so they did not see the need to adhere to their ART regimens. Others felt hopeless and not motivated to adhere to their ART regimens. A few others reported that because ART caused unwanted changes to their body, they were reluctant to adhere to the regimen. Lastly, the size of the pill hindered adherence and that substance use takes priority over ART adherence was reported by a few participants.

## Discussion

To our knowledge this is the first in-depth qualitative study on adherence facilitators and barriers of ART medication adherence among people living with HIV in Lima, Peru. Our study identified that having a fixed routine, understanding the need for compliance, seeing positive results, knowledge of treatment, faith in treatment, and use of reminders tools were the most frequently cited facilitators. In addition, side effects, simply forgetting, inconvenient schedule, financial constraints, being away from home, and fear of disclosure/stigma were the most frequent cited barriers.

The results of this study are similar to those of other qualitative studies in developing countries [28]. For example, in a study conducted in Brazil, Brigido et al. (2001) reported that forgetfulness and intolerance were the most frequent reasons for non-adherence. Other reasons cited were stopping ART to consume alcohol, misunderstanding of the prescription requirements, difficulty in following recommendations at the workplace, and lack of money for transportation to obtain medication [36]. In Botswana, Weiser et al. (2003) found that the principal barriers to HIV adherence were financial constrains, stigma, travel/migration, and side effects [37]. In our study, we did not find that migration was a barrier. In Uganda, Byakika-Tusiime (2005) et al. reported that shortage of drugs due to lack of money was the most common reason for non-adherence. Other reasons included forgetfulness, drug inaccessibility, adverse effects of the drugs, travelling away from home, unclear instructions by the health provider, and being too busy [38]. In another study conducted in Brazil, Pinheiro et al. (2002) found that self-efficacy expectation, the perception of negative effects, and physical concerns were associated with adherence [39].

Similarly, in a qualitative study conducted with Spanish-speaking patients in Los Angeles, Murphy et al. (2003) reported that the most frequent barriers for non-adherence were feeling depressed or feeling overwhelmed, simply forgot, and timing difficulties. Our study, however, did not find feeling depressed/stressed as a common barrier. In the same study, the most frequently identified adherence strategies were making an effort to learn more about the antiretroviral medications, accepting the need to take antiretroviral medications, and refilling prescriptions early or on time [40].

Lastly, a number of studies have found that patients with a trusting and open relationship with their health care providers are better able to adhere to their ART regimens [28]. Furthermore, patients who lack open communication with their health care providers due to cultural differences, cultural insensitivity, and/or language barriers may find it difficult to adhere to ART regimens [41].

Effective interventions to improve adherence to ART are able to build on adherence facilitators and address adherence barriers. For example, one study at 6 HIV specialty clinics in California that included 437 HIV positive participants, it was demonstrated that participants who participated in a clinic-wide intervention included a brief counselling session with their medical provider that established open communication, built trust, and addressed obstacles to complying with pill taking regimens were more likely to remain adherent to ART [42]. In a review of behavioral interventions that promote ART adherence, Simoni et al. (2008) found that there are a number of specific strategies that may be effective at promoting adherence provided that they are culturally sensitive and address structural and individual level barriers. These strategies include solidifying the patient-provider relationship by establishing trust, promoting social support, employing simple practical strategies, managing side effects, and addressing misinformation related to adherence practices [43].

In Lima, HIV positive individuals may be in particular need of ART adherence interventions that address strategies to improve adherence through the patient-provider relationship and improve strategies to incorporate ART pill taking regimens into a busy urban lifestyle where individuals may feel stigmatized by their HIV positive status. One effective strategy that may address these factors in an environment such as Lima, Peru may involve the use of cell phones. Cell phones have been successfully used to support patient medication adherence in developed countries [44] and in resource-constrained settings such as Uganda [45], and South Africa [46]. In Peru, Curioso et al. are currently evaluating a one-year randomized trial, Cell-POS http://www.cellpos.org, a computer-based intervention using cell phones (via short text messages) to enhance adherence to antiretroviral treatment (ART) and support HIV care among adult HIV- positive patients. Cell phones might be an ideal tool to improve ART adherences for HIV patients in Lima because they can be private, interactive, efficient, affordable, convenient, and useful as a reminder tool [47,48].

Our study was limited by its use of a convenience sample of people living with HIV/AIDS in an urban population of Lima. The participants in this study cannot be considered representative of people living with HIV/AIDS throughout Peru. However, one of the strengths of this study is that we are confident that we reached saturation because a number of the themes were similar among participants.

Additional qualitative and quantitative studies are needed in Peru that further explore the barriers and facilitators of ART adherence for women and other demographic groups in Lima and for Peruvians living in other rural and urban regions of Peru. Specifically, more research is need on strategies to improve adherence for sex workers who experience high rates of HIV infection. In addition, more research is needed to understand what mechanisms support the transition from managing the ART regimen and side effects to successfully internalizing self-care to achieve high levels of ART adherence. Multi-component, tailored intervention programs that specifically address the barriers of ART adherence and build on the facilitators of ART adherence in Peru should be developed and pilot tested at clinics and health care facilities that provide ART. Lastly, in Peru, more widespread public health education campaigns are needed to decrease HIV/AIDS stigma and to increase community acceptance of individuals living with HIV/AIDS. Additional public health programs are needed to increase levels of direct interpersonal social support networks for individuals living with HIV who are on ART.

## Conclusions

Overall, these findings were similar to the facilitators and challenges experienced by individuals on ART in other resource constrained settings. However, this is the first study to use qualitative methods to investigate factors related to ART adherence in Lima, Peru. The findings from our study can be used to inform the development of effective interventions that address the barriers and facilitators of ART adherence in Peru. Specifically, highlighting positive health results due to the ART treatment regimen with patients, developing effective reminder tools, and promoting faith in treatment may improve levels of ART adherence in Lima. Furthermore, helping patients to physically and emotionally manage ART side effects, to improve strategies for incorporating ART regimens into their daily lives, and for alleviating financial constraints to adherence to ART will also improve long term ART adherence for HIV positive patients in Lima.

## Competing interests

The authors declare that they have no competing interests.

## Authors' contributions

WHC conceived the study, collected the data, and led the analysis, interpretation, and manuscript drafting. DK participated in the data analysis and helped draft the manuscript. AK conceived the study, made substantial contributions to the data analysis, interpretation of data, and helped draft the manuscript. RC and PS reviewed the manuscript and made significant comments. All authors read and approved the final manuscript.

## Pre-publication history

The pre-publication history for this paper can be accessed here:

http://www.biomedcentral.com/1471-2458/10/13/prepub
